# Dangling Choroid Plexus: An Early Sign of Fetal Anomaly

**DOI:** 10.3390/diagnostics16091302

**Published:** 2026-04-27

**Authors:** Anastasija Arechvo, Michael Brusilov, Antigoni Hadjiiona, Gustavo Malinger, Karina Krajden Haratz, Kypros H. Nicolaides

**Affiliations:** 1 Fetal Medicine Research Institute, King’s College Hospital, London SE5 8BB, UK; 2 Institute of Women and Children’s Health, School of Life Course and Population Sciences, King’s College London, London SE1 7EH, UK; 3 Obstetrics and Gynecology Ultrasound Unit, Lis Maternity Hospital, Tel Aviv Sourasky Medical Center, Tel Aviv 6971068, Israel; brusilovm@gmail.com (M.B.);; 4 Grey Faculty of Medicine, Tel Aviv University, Tel Aviv 6997801, Israel

**Keywords:** ultrasound, neurosonography, genetic testing, choroid plexus, brain pathology

## Abstract

**Objectives**: This study aimed to examine the association between the dangling choroid plexus sign and fetal structural, chromosomal, and genetic abnormalities, as well as to define the normal range of lateral ventricular width and the ratio of choroid plexus width to lateral ventricular width at 14–17 weeks of gestation. **Methods**: This retrospective study analyzed ultrasound images from early fetal anatomy scans performed between January 2018 and July 2025 at two tertiary fetal medicine centres. In centre A, 6063 singleton pregnancies underwent routine scans at 11–13 and 14–17 weeks. In centre B, 776 fetuses with suspected abnormalities or increased nuchal translucency at 11–13 weeks were reassessed at 14–17 weeks. Additionally, 400 fetuses without obvious abnormalities at 14–17 weeks were used to determine normal ventricular measurements. **Results**: In normal fetuses, the mean lateral ventricular width was 6.90 mm (95% CI 6.81–6.99) and the mean choroid plexus-to-ventricle ratio was 0.85 (95% CI 0.84–0.86). A dangling choroid plexus was identified in 38 fetuses (0.16% in routine and 3.6% in high-risk populations). Out of 38 cases of dangling choroid plexus, 37 were associated with additional structural defects, chromosomal abnormalities, or single-gene disorders. Chromosomal abnormalities were found in 11/30 tested cases, most commonly trisomy 21. The most common defects observed on initial or subsequent scans were ventriculomegaly, cardiac defects, and abnormal posterior fossa. **Conclusions**: A dangling choroid plexus at 14–17 weeks is a sonographic marker associated with major fetal abnormalities and should prompt detailed anatomical assessment and consideration of genetic testing.

## 1. Introduction

Ventriculomegaly, defined as an atrial width of ≥10 mm, is a well-established sonographic marker associated with a broad spectrum of fetal cranial and extracranial anomalies when identified during the mid-trimester ultrasound examination [1]. Despite extensive literature addressing ventriculomegaly in the second and third trimesters [2,3], there remains a relative paucity of standardized diagnostic criteria for identifying abnormal ventricular development during the late first and early second trimesters of pregnancy.

**Figure 1:**
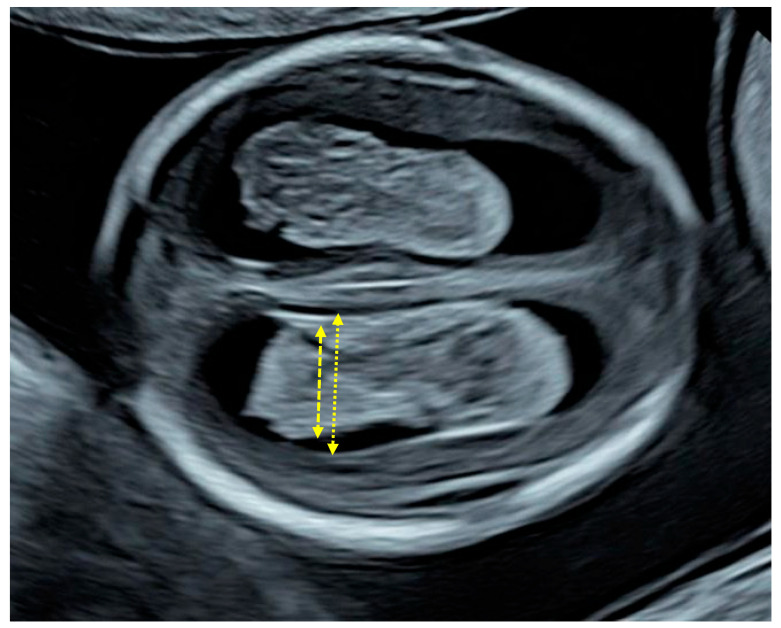
Demonstration of the measurement of the choroid plexus by placing the callipers at the most distal edges of the plexus in the same transverse view used for the measurement of the lateral ventricles.

During early gestation, particularly between 11 and 14 weeks, the normal sonographic appearance of the fetal brain is characterized by the choroid plexus occupying the majority of the lateral ventricles [4,5] (Figure 1). This results in a “filled” appearance, where the plexuses closely oppose the ventricular walls, leaving minimal visible cerebrospinal fluid (CSF) space. This developmental stage reflects rapid neuroepithelial growth and the prominent role of the choroid plexuses in CSF production during early brain formation [6]. A deviation from this pattern, specifically, rather than filling the ventricle, the plexuses appear reduced in size relative to the ventricular space, thereby creating a visible gap between the plexuses and the ventricular walls. This finding is thought to reflect an imbalance between ventricular expansion and choroid plexus growth, potentially indicating early ventricular dilatation or impaired neurodevelopment [1,4,7].

Previous studies have highlighted the significance of this finding in early gestation. A cohort study of 17 fetuses that were diagnosed with ventriculomegaly at mid-gestation reported that at 11–14 weeks, the choroid plexus to the lateral–ventricle ratio was significantly lower than in 100 fetuses without ventriculomegaly [8]. Despite these observations, there is limited data regarding the significance of a dangling choroid plexus beyond the first trimester, particularly in the early second trimester. Furthermore, normative data describing the relationship between choroid plexus size and ventricular dimensions at 14–17 weeks of gestation are lacking. Establishing such reference ranges is essential to distinguish physiological variation from pathological findings.

The present study aims to evaluate the association between the presence of a dangling choroid plexus at 14–17 weeks of gestation and the occurrence of fetal structural abnormalities, chromosomal anomalies, and genetic disorders and to establish normative reference ranges for lateral ventricular width and the ratio of the choroid plexus width to ventricular width during the early second trimester development.

## 2. Methods

### 2.1. Study Population and Design

This was a retrospective cohort study of fetuses examined in two centres between January 2018 and July 2025. In one centre, Lis Maternity Hospital, Tel Aviv, Israel, 6063 women with singleton pregnancies, who booked for pregnancy care and delivery, attended a routine scan at 11 + 0 to 13 + 6 weeks of gestation and again at 14 + 0 to 17 + 6 weeks. The 11–13 weeks scan was mainly aimed at the measurement of fetal crown–rump length (CRL) [9], nuchal translucency thickness (NT), and the detection of major defects [10], whereas the 14–17 weeks scan, which was carried out transabdominally and transvaginally, included a detailed and systematic examination of fetal anatomy [11,12]. In the second centre, King’s College Hospital, London, UK, a routine transabdominal scan was carried out at 11–13 weeks and, in addition to the measurements of fetal CRL and NT, a systematic examination of fetal anatomy was performed. In 776 cases with a suspected defect or increased NT, a transabdominal and transvaginal scan was repeated at 14–17 weeks. In both centres, gestational age was determined from the crown–rump length at the 11–13-week scan. We included all cases with the dangling choroid plexus but excluded cases with spina bifida.

The assessment of a “dangling” choroid plexus was performed qualitatively based on its sonographic appearance rather than using a predefined quantitative cut-off value. Specifically, the diagnosis was made subjectively by the examiner when the choroid plexus was observed to hang dependently within the ventricular cavity, separated from the ventricular wall, in keeping with established descriptive criteria.

At the 14–17-week scan, the width of the distal lateral cerebral ventricle was measured in a transverse view at the level of the atrium. Transvaginal sonography was carried out for a detailed examination of the fetal anatomy and, depending on the findings, chorionic villous sampling or amniocentesis was carried out for fetal karyotyping and microarray analysis. In cases where karyotyping and microarrays were reported as normal but ultrasound findings raised suspicion of an underlying genetic abnormality, whole-exome sequencing was performed whenever possible.

For the purposes of this study, we retrieved from the clinical database patient data and images and retrospectively measured the width of the choroid plexuses by placing the callipers at the most distal edges of the plexus in the same transverse view used for the measurement of the distal lateral ventricles (Figure 1).

In the second centre, King’s College Hospital, London, UK, we also retrieved patient data on 400 fetuses from women who presented as late bookers for the first ultrasound examination at 14–17 weeks of gestation with no obvious fetal abnormalities. We retrospectively measured the width of the lateral ventricle and choroid plexus by placing the callipers at the most distal edges of the plexus in the same transverse view used for the measurement of the lateral ventricle.

Data on prenatal findings, pregnancy outcomes, and postnatal diagnoses were collected from hospital records. In the UK, this study constitutes an analysis of data derived from routine clinical examinations and therefore did not require ethics committee approval. In Israel, approval for this study was obtained from the institutional review board (IRB #TLV-0109-26).

### 2.2. Statistical Analysis

Continuous variables are presented as the median (range) and qualitative variables are presented as absolute and relative frequencies (%). The Mann–Whitney U test was used for the comparison of the continuous variables between the groups. To examine the association between gestational age and each outcome variable, generalized linear models (GLMs) were fitted, beginning with linear terms and expanded to quadratic and cubic polynomial models to assess potential non-linear relationships. In addition, B-splines and natural cubic splines were applied to flexibly model the non-linear effects of gestational age. Percentile estimates (5th, 10th, 25th, 50th, 75th, 90th, and 95th) for the choroid plexus to distal lateral ventricle ratio were obtained using quantile-based calculations. All statistical analyses were conducted using R 4.5.3 (R Foundation for Statistical Computing, Vienna, Austria).

## 3. Results

### 3.1. Study Population

In the cohort from Israel of 6063 routine early second trimester scans, 10 fetuses (0.16%) were identified as having a dangling choroid plexus. Notably, all of these cases had normal findings at the earlier 11–13-week scan, including normal NT measurements and no detectable structural abnormalities. However, abnormalities became apparent at the 14–17-week scan.

In the UK cohort, which included 776 higher-risk cases, 28 fetuses (3.6%) were found to have a dangling choroid plexus. The higher prevalence in this group likely reflects the pre-selection of cases with suspected abnormalities or increased NT.

### 3.2. Measurements of Lateral Ventricles and Choroid Plexus in Normal Fetuses

**Figure 2:**
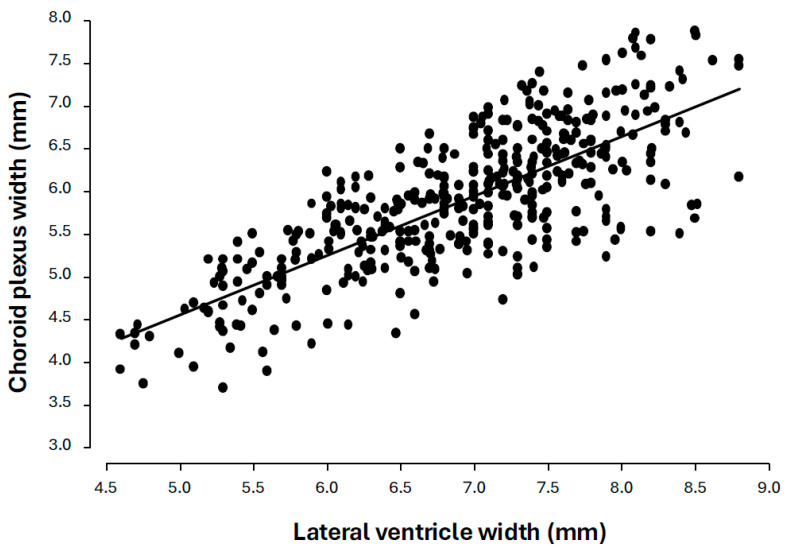
Individual measurements and regression line of the relation of the width of the lateral ventricle to the size of the choroid plexus in normal fetuses at 14–17 weeks of gestation.

**Figure 3:**
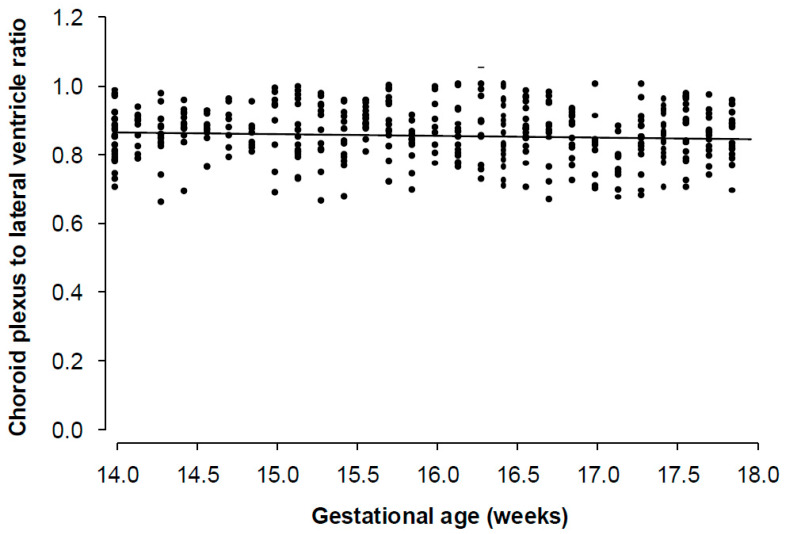
Individual measurements and regression line of the relation of the choroid-plexus-to-lateral-ventricle ratio with gestational age in normal fetuses.

In the 400 normal cases at 14–17 weeks of gestation, the width of the lateral ventricle did not change significantly with gestation; the mean was 6.90 mm (95% CI 6.81–6.99 mm). Similarly, the width of the choroid plexus did not change significantly with gestation; the mean was 5.88 mm, and the 95% CI was 5.80 to 5.96 (Figure 2). The ratio of the width of the choroid plexus to that of the lateral ventricle was 0.85 and the 95% CI was 0.84–0.86 (Figure 3). In the normal cases, the centiles of the ratio were as follows: 5% (0.71), 10% (0.74), 25% (0.8), 50% (0.86), 75% (0.90), 90% (0.95), and 95% (0.97) (Table 1). Across all fitted models, including linear, quadratic, cubic polynomial, B-spline, and natural spline formulations, gestational age did not emerge as a significant predictor of ventricular volume, choroid plexus volume, or the choroid-plexus-to-lateral-ventricle ratio.

### 3.3. Measurements of Lateral Ventricle and Choroid Plexus in Fetuses with Dangling Choroid Plexuses

In the combined data of 38 cases of dangling choroid plexuses, the mean gestational age at the time of the early anatomy scan was 16 + 4 weeks (range 14 + 0 to 17 + 6 weeks), and the mean head circumference was 128.9 mm (range 87.0–159.0 mm). The median width of the lateral ventricle was 9.4 mm and the range was 6.7 mm to 15.8 mm. Similarly, the width of the choroid plexus did not change significantly with gestation; the median was 4.8 mm and the range was 2.6 to 7.8. The ratio of the width of the choroid plexus to that of the lateral ventricle was 0.5 and the range was 0.2–0.9. All three variables were significantly different from the group without pathologies, *p* < 0.001.

### 3.4. Fetal Abnormalities

In 37 of the 38 cases with dangling choroid plexus, there were additional anatomical defects, noticed on the scan at 14–17 weeks of gestation or on the subsequent scans, chromosomal abnormalities, or single-gene disorders (Table 2).

**Figure 4:**
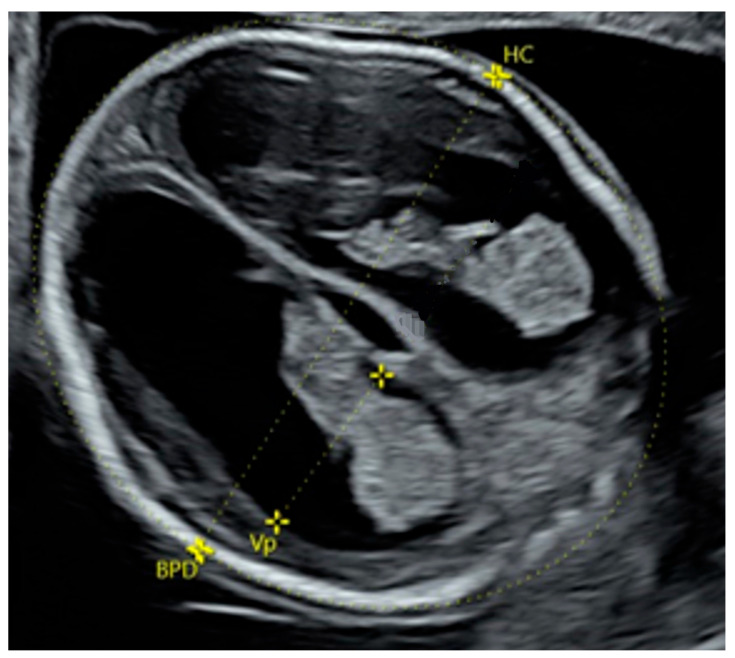
Dangling appearance of the choroid plexus at 15 + 6 weeks of gestation. BPD, biparietal diameter; HC, head circumference.

**Figure 5:**
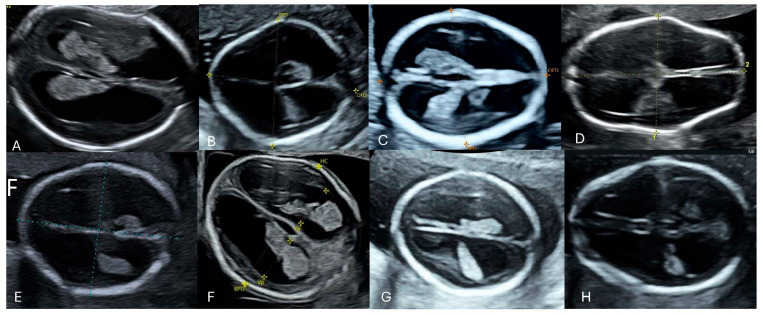
Dangling appearance of the choroid plexuses between 14 and 17 weeks of gestation. (**A**) 15 + 4 weeks of gestation; (**B**) 16 + 5 weeks of gestation; (**C**) 15 + 1 weeks of gestation; (**D**) 17 + 0 weeks of gestation; (**E**) 17 + 1 weeks of gestation; (**F**) 15 + 6 weeks of gestation; (**G**) 17 + 2 weeks of gestation; (**H**) 16 + 6 weeks of gestation.

**Figure 6:**
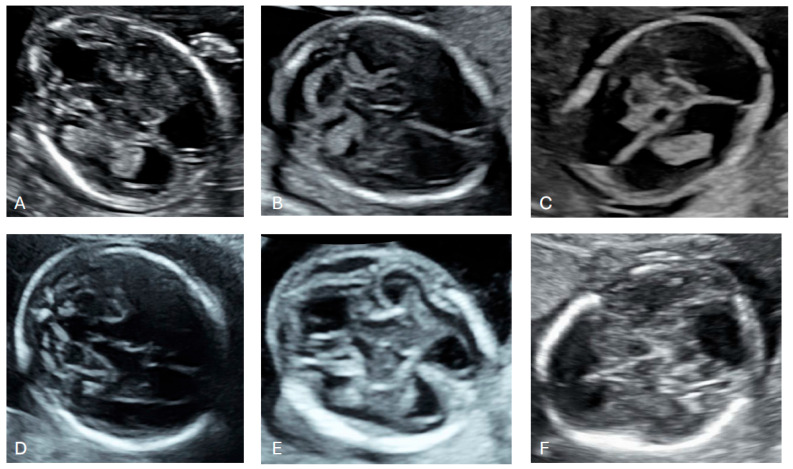
Cases with abnormal posterior fossa. (**A**) 15 + 2 weeks of gestation, vermian agenesis; (**B**) 16 + 2 weeks of gestation, cerebellar hypoplasia; (**C**) 14 + 0 weeks of gestation, absent cerebellum and vermis; (**D**) 17 + 0 weeks of gestation, vermian hypoplasia; (**E**) 15 + 6 weeks of gestation, cystic dilatation of the fourth ventricle; (**F**) 16 + 2 weeks of gestation, agenesis of the cerebellum and vermis.

**Figure 7:**
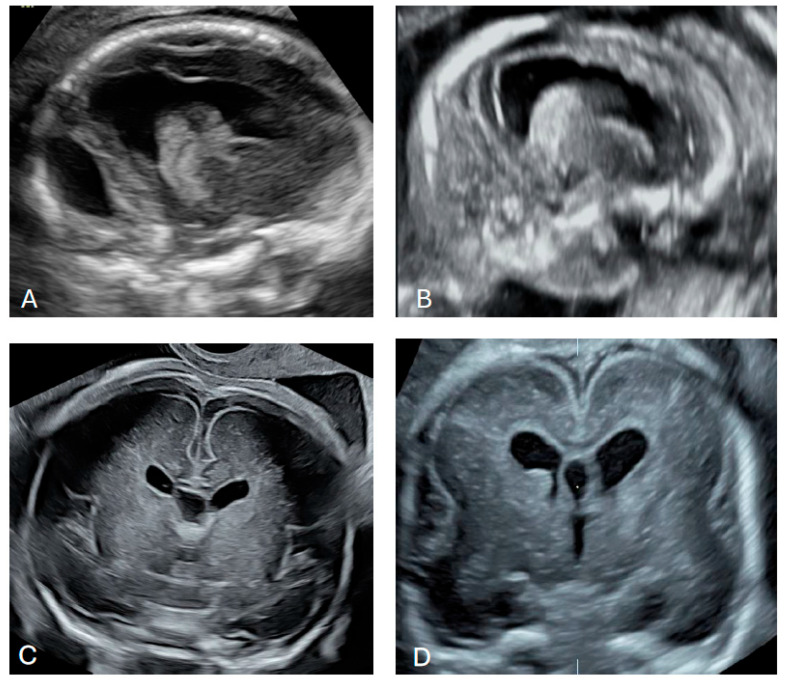
Cases with abnormal cortical development. (**A**) 18 + 1 weeks of gestation, irregular ventricular wall; (**B**) 17 + 4 weeks of gestation, irregular ventricular wall suggesting periventricular nodular heterotopia; (**C**) 31 + 6 weeks of gestation, delayed cortical maturation; (**D**) 21 + 3 weeks of gestation delayed operculisation of the Sylvian fissure, abnormal germinal matrix.

In 38 cases, the abnormal size or shape of the lateral ventricle was clearly identified even before making measurements (Figure 4 and Figure 5). Associated abnormalities were detected at the time of the 14–17-week scan or in subsequent examinations. The most common anomaly was ventriculomegaly, which was observed in 15 (39.5%) cases on subsequent scans. Only in one case was ventriculomegaly an isolated finding. The second most common anomalies were cardiac defects observed in 12 (31.6%) cases. An abnormal posterior fossa was observed in eight (21.1%) cases (Figure 6). Malformations of cortical development were observed in five (13.2%) cases, including periventricular nodular heterotopia and delayed sulcation (Figure 7). Other anomalies, found in 1–2 cases each, included cataract, cleft palate, skeletal dysplasia, caudal regression syndrome, talipes, abnormal kidneys, early fetal growth restriction, and hydrops fetalis.

Fetal karyotyping and microarrays were carried out in 30 cases and chromosomal abnormalities were identified in 11 (36.7%); this included trisomy 21, trisomy 18, trisomy 13, mosaic trisomy 17, 1q21 mosaicism, and triploidy. In five cases, whole-exome sequencing was performed, and a single-gene disorder (*TUBB2A*, *COL1A1*, and *OFD1* gene mutations) was detected in three cases.

### 3.5. Pregnancy Outcomes

In 34 of the 38 cases with dangling choroid plexuses, pregnancy termination was performed at the request of the parents. Of the remaining four cases, ventriculomegaly resolved spontaneously in two, one fetus presented with growth restriction and cataract, and one showed no additional antenatally detected ultrasound anomalies apart from the dangling choroid plexus. All four pregnancies resulted in live births, and the infants demonstrated apparently normal postnatal development.

## 4. Discussion

### 4.1. Main Findings

In normal pregnancies at 14–17 weeks of gestation, the width of the lateral ventricle did not change significantly with gestation; the mean is 6.90 mm (95% CI 6.81–6.99) and the mean ratio of the width of the choroid plexus to that of the lateral ventricle is 0.85 (95% CI 0.84–0.86). However, the ultrasound finding of a dangling choroid plexus may indicate an underlying structural, chromosomal, or genetic abnormality.

A dangling choroid plexus was identified in 10 fetuses (0.16%) in the routine early second-trimester screening cohort, whereas 28 fetuses (3.6%) in the high-risk cohort were found to have this. We acknowledge that the inclusion of both a routine screening population and a high-risk referral cohort may introduce spectrum bias, which can influence the observed incidence and clinical performance of the studied marker. Therefore, the findings should be interpreted with caution when considering overall prevalence and potential predictive implications.

In our cohort of 38 affected fetuses, the median choroid plexus-to-lateral ventricle ratio was markedly reduced to 0.5 (range 0.2–0.9) (*p* < 0.001), indicating a clear disproportion between these structures. Although the ratio of choroid plexus to ventricular width differed significantly between groups, the observed overlap in values precludes the definition of a reliable diagnostic cut-off, supporting the interpretation of a dangling choroid plexus as a qualitative sonographic sign rather than a quantitative marker.

The observed disproportion likely reflects early disturbances in normal cerebral development, relative ventricular enlargement, impaired development of periventricular brain structures, or reduced choroid plexus volume. Such changes may be linked to alterations in cerebrospinal fluid dynamics or underlying genetic and developmental abnormalities.

### 4.2. Comparison with Results of Previous Studies

Previous studies have primarily focused on ventriculomegaly detected later in pregnancy, typically after 20 weeks of gestation or even in the third trimester, when ventricular dilatation becomes more apparent [13,14,15]. In contrast, our findings suggest that the visual disproportion between the lateral ventricle and choroid plexus may provide a subtle but reliable early sign of cranial and extracranial pathologies. This aligns with the anatomical expectation that in early pregnancy, before 20 weeks of gestation, a normal choroid plexus should fill the lateral ventricles [4,5]. With advancing gestational age, the choroid plexus typically shifts posteriorly along the choroidal fissure, behind the foramen of Monro [16]. Consequently, an apparent separation or “dangling” configuration of the choroid plexus earlier in pregnancy deviates from this expected pattern and may reflect early disruption in ventricular development, cerebrospinal fluid dynamics, or global brain growth.

Previous studies have demonstrated that ventriculomegaly can indicate structural brain pathology [7,17,18]. Our findings suggest that early identification of a dangling choroid plexus may serve as an early marker of brain pathology, as in our cohort, it preceded the development of ventriculomegaly, posterior fossa abnormalities, agenesis of corpus callosum, and cortical developmental disruptions.

In our cohort, the sonographic finding of a dangling choroid plexus was also associated with a high incidence of chromosomal abnormalities and genetic syndromes. Ultrasonographic studies at 11–14 weeks of gestation reported that a decrease in the size of the choroid plexus, in comparison to the size of the lateral ventricles, is associated with aneuploidy [7]. Moreover, the presence of a dangling choroid plexus may indicate other genetic disorders, including single-gene syndromes, underscoring its potential as a marker of underlying developmental pathology. Given that ventriculomegaly later in pregnancy is recognized as a feature of diverse genetic conditions [19], early identification of a dangling choroid plexus at 14–17 weeks of gestation can expedite targeted genetic evaluation and optimize prenatal counselling.

### 4.3. Implications for Clinical Practice

The presence of a dangling choroid plexus, irrespective of the ventricular width, may represent an easily recognizable and clinically important early sonographic marker that may be associated with a spectrum of intracranial as well as extracranial fetal abnormalities. Importantly, this sign may precede the development of overt ventriculomegaly, thereby offering an opportunity for earlier identification of fetuses at risk. Its detection should not be interpreted in isolation but rather as an indication for a thorough and systematic fetal assessment, including a detailed transvaginal evaluation of the central nervous system and other organ systems. In addition, given the reported association with chromosomal and genetic conditions, consideration should be given to invasive diagnostic testing to enable comprehensive genetic analysis where clinically appropriate. In contrast, observation of the choroid plexus filling the lateral ventricle at 14–17 weeks of gestation should be a reassuring finding.

Looking ahead, advances in artificial intelligence-assisted fetal neuroimaging are expected to further enhance the detection and characterization of subtle intracranial markers such as the dangling choroid plexus. Automated or semi-automated image analysis tools may improve measurement consistency, reduce interobserver variability, and facilitate earlier and more standardized recognition of abnormal ventricular morphology, thereby supporting clinical decision-making and risk stratification in routine prenatal care [20,21].

### 4.4. Strength and Limitations

The strengths of this study are first that it involves a large cohort from two specialist fetal medicine centres; second, this is the first study describing abnormal choroid plexus in the population of fetuses examined at 14 + 0 to 17 + 6 weeks of gestation; third, in all cases, a detailed fetal examination was carried out by experts in fetal medicine and in most cases, genetic studies were performed; and fourth, the normal range of the width of the lateral ventricle and ratio of the width of the choroid plexus to that of the lateral ventricle at 14–17 weeks of gestation was established.

There are several limitations of the study, including that it uses a retrospective design, and the potential for underreporting anomalies is not evident until later gestation or postnatally; second, the assessment of choroid plexuses was based on subjective descriptions from specialists and were based on qualitative sonographic interpretation rather than a standardized or quantitative definition, which may introduce inter-operator variability and potential selection bias; third, the high rate of pregnancy terminations in our cohort may have limited the assessment of the natural history of this finding and introduced potential underreporting of anomalies that might only become apparent later in gestation or postnatally; and fourth, while short-term postnatal outcomes were reassuring, long-term follow-up data were not available for the included cases. Future prospective studies with extended follow-up are needed to better determine the prognostic significance of these findings.

## 5. Conclusions

A dangling choroid plexus at 14–17 weeks of gestation may be considered a potentially important sonographic marker suggestive of significant underlying fetal abnormalities. Incorporating a detailed assessment of the choroid plexus and ventricular width in early anatomy protocols may facilitate earlier diagnosis and intervention.

## Figures and Tables

**Table 1:** Centile distribution of the ratio in normal cases.

Centile (%)	Ratio
5	0.71
10	0.74
25	0.80
50 (median)	0.86
75	0.90
90	0.95
95	0.97

**Table 2:** Ultrasound findings and results of genetic studies in the 38 fetuses with dangling choroid plexus at 14 to 17 weeks of gestation.

Ultrasound Findings	NT (mm)	Genetic Studies	Outcome
Abnormal posterior fossa	2.6	1q21 moscaicism	TOP
Abnormal posterior fossa	1.7	Normal	TOP
Abnormal posterior fossa, AVSD	1.9	Normal	TOP
Abnormal posterior fossa, CoA, short long bones, dysplactic kidneys	1.2	Mosaic trisomy 17	TOP
Abnormal posterior fossa, echogenic kidneys	1.8	No	TOP
Abnormal posterior fossa, postaxyal polydactyly	1.4	OFD1 gene mutation	TOP
Abnormal posterior fossa, ventriculomegaly *	1.5	TUBB2A gene mutation	TOP
Abnormal posterior fossa, ventriculomegaly *, cortical malformation *	0.7	Normal	TOP
Absent corpus callosum, cortical malformation, talipes	3.5	Normal	TOP
AVSD	0.9	Normal	TOP
AVSD, cleft palate, caudal regression syndrome	2.5	Normal	TOP
AVSD, ventriculomegaly *	1.4	Normal	TOP
Cleft palate	1.0	No	TOP
Clenched hands, VSD, overriding aorta, ventriculomegaly *	1.4	Trisomy 18	TOP
Clover-shaped head, severe shortening of limbs, micrognathia, talipes	1.5	COL1A1 gene mutation	TOP
Cortical malformation	1.6	No	TOP
Dangling choroid plexus	0.9	No	Alive
Dangling choroid plexus	1.7	Trisomy 21	TOP
Dangling choroid plexus	1.8	No	TOP
DORV, PA, ARSA, hydronephrosis	2.0	Normal	TOP
Encephalocele, ventriculomegaly *	3.8	Normal	TOP
FGR	1.3	Trisomy 13	TOP
FGR, cataract	1.0	No	Alive
FGR, CoA, ventriculomegaly *	1.9	Normal	TOP
FGR, major cardiac defect	1.7	Triploidy	TOP
FGR, ventriculomegaly *, cortical malformation *	2.1	Normal	TOP
Generalised edema, hydrothorax, ascites	2.8	Trisomy 21	TOP
Hydronephrosis, LUTO, ventriculomegaly *, **	1.6	Normal	Alive
Hyperechogenic bowel	3.0	Trisomy 21	TOP
Hyperechogenic bowel, mild pericardial effusion, hypoplastic nasal bone	1.8	Trisomy 21	TOP
Increased NF, absent nasal bone, ARSA	4.8	Trisomy 21	TOP
Increased NF, ventriculomegaly *	3.2	Normal	TOP
Iniencephaly	6.9	Normal	TOP
Pulmonary stenosis, ventriculomegaly *, cortical malformation *	5.9	Normal	TOP
Talipes, ventriculomegaly *	1.6	Normal	TOP
Ventriculomegaly *, absent corpus callosum *	2.5	Trisomy 21	TOP
Ventriculomegaly *, absent corpus callosum *	3.2	No	TOP
Ventriculomegaly *, **	1.5	No	Alive

ARSA, aberrant right subclavian artery; AVSD, atrioventricular septal defect; CoA, coarctation of the aorta; DORV, double outlet right ventricle; FGR, fetal growth restriction; LUTO, lower urinary tract obstruction; NT, nuchal translucency; PA, pulmonary atresia; TOP, termination of pregnancy; VSD, ventricular septal defect. * Subsequent scan. ** Ventriculomegaly has resolved on later stage of pregnancy.

## Data Availability

The data presented in this study are available on request from the corresponding author.
